# Effects of Simulation-Based Learning on Nursing Students’ Learning Outcomes of a Gerontology Course

**DOI:** 10.1093/geroni/igab046.1880

**Published:** 2021-12-17

**Authors:** Hsueh-Fen Kao, Minzhi Ye, Lin Chen

**Affiliations:** 1 The University of Texas at El Paso, El Paso, Texas, United States; 2 Benjamin Rose Institue on Aging, Benjamin Rose Institue on Aging, Ohio, United States; 3 Fudan University, Shanghai, Shanghai, China (People's Republic)

## Abstract

Simulation-Based Learning (SBL) is beneficial to nursing education. Nevertheless, recent studies have shown a side effect of being overwhelmed by repeated exposures to simulation. Thus, how many times simulation scenarios should be provided to students remains a question. The objectives of this study were to (1) explore the changes in nursing students’ perceived competence, self-efficacy, and learning satisfaction after repeated exposures to simulations, and (2) determine the acceptable frequency of SBL in the ‘Care of Older Adults’ course. A one-group repeated measurement experimental design with self-administered questionnaires in a convenient sample of 84 senior nursing undergraduate students was used at a university in southern Taiwan, and 79 students completed all measurements. After taking the baseline measurements (T0), students were exposed to 75-mininute simulation scenarios from Time 1 (T1) to Time 3 (T3) three weeks apart throughout the semester. Students’ perceived nursing competence, self-efficacy, and learning satisfaction were measured immediately after each exposure. There were statistically significant improvements from T0 to T3 (p < .001) in all three areas; however, no significant difference when comparing scores from T1 to T2 and from T2 to T3. To conclude, SBL is effective in improving nursing students’ perceived competence, self-efficacy, and learning satisfaction. While the primary changes occur at the first simulation effort, it is the accumulated multiple exposures collectively improve students’ learning outcomes. Multiple instructional strategies are recommended to maintain students’ learning interests to achieve optimal learning outcomes of the course across a semester.

